# Anterior Cruciate and Anterior Oblique Ligament Reconstruction Using Hamstrings and Peroneus Longus’ Anterior Half Grafts

**DOI:** 10.1016/j.eats.2023.02.019

**Published:** 2023-05-08

**Authors:** Pedro Baches Jorge, Melanie Mayumi Horita, Marconde de Oliveira e Silva, Sérgio Marinho de Gusmão Canuto, Camilo Partezani Helito, Diego Escudeiro de Oliveira

**Affiliations:** a Department of Orthopedics and Traumatology, Faculdade de Ciências Médicas da Santa Casa de Misericórdia de São Paulo, São Paulo, Brazil; b Santa Casa de Misericórdia Hospital de Maceió, Maceió, Alagoas, Brazil; c Department of Orthopedics and Traumatology, Faculty of Medicine, University of São Paulo, São Paulo, Brazil

## Abstract

Anterior cruciate ligament injuries are common in high school and college with an estimated 120,000 cases per year in the United States. Most sports injuries occur without direct contact, and knee valgus with external rotation of the foot is the most common movement. This movement may be related to the injury of the anterior oblique ligament located in the anteromedial quadrant of the knee. This technical note presents anterior cruciate ligament reconstruction with extraarticular anteromedial reinforcement using hamstring and the anterior half of the peroneus longus grafts.

## Introduction

More than 120,000 anterior cruciate ligament (ACL) injuries occur every year in the United States, mostly during the high school and college years.^1^ The incidence of these injuries is slowly increasing, and the return to sport in the same level is not great.^1^ The role of concomitant extra-articular procedures in improving the outcome of ACL reconstruction has experienced a recent resurgence in interest.^2^

The use of hamstring tendons in ACL reconstruction is widely accepted, and the results comparing with the patellar tendon, even in athletes, are the same,^3^ and numerous studies have described the peroneus longus (PL) as a graft that has adequate biomechanical properties and is safe to use in ACL reconstruction (ACLR) without major biomechanical and kinematic repercussions for the foot and ankle from which it was harvested.^4^

The most ACL lesions in sports occur without direct trauma, and the valgus knee and foot external rotation are the most common biomechanical movements.^5^ Further, the anteromedial rotational instability may have an important role in the success of the ACL treatment. This is the reason that the anteromedial compartment, responsible for valgus and external rotational control,^6^ gains importance.

The anterolateral ligament (ALL) is a distinct structure in the anterolateral portion of the knee. After the study of Jorge et al.,^6^^,^^7^ a similar ligament structure in the anteromedial knee quadrant is known: the anterior oblique ligament (AOL). It is possible that in specific cases of ACL ruptures, like the history of valgus and external rotational traumas, partial anteromedial peripherical lesions in MRI, or mild medial instability, the anteromedial reinforcement can lead to good results.

## Surgical Technique

The procedure is performed with the patient in the supine position on the surgical table. A pneumatic tourniquet is placed proximally on both lower limbs and inflated after the placement of sterile drapes.

### Hamstring Tendon Harvesting

Harvest of the tendons of the semitendinosus and gracilis (STG) muscles is preferentially performed in the injured limb. First, a longitudinal incision of approximately 3 cm is made 4 cm distal to the knee joint. A new transverse incision is made in the fascia of the sartorius muscle, and the STG is isolated. The STG is repaired with no. 1-0 Vicryl thread (Ethicon, Somerville, NJ), and its proximal insertions are removed with the aid of a tenotome.

### Anterior Half Peroneus Longus Harvesting

**Fig 1:**
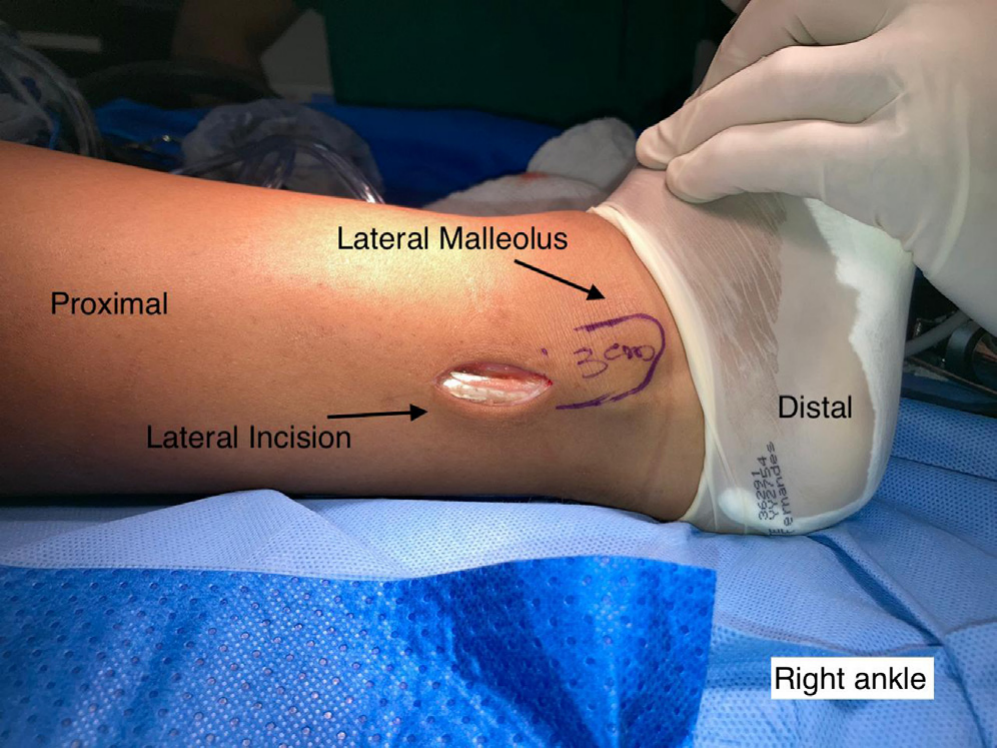
This photo shows the right ankle being prepared for removal of the peroneus longus tendon graft. A line delimiting the lateral malleolus was drawn. An incision was made starting 3 cm proximal to the most distal point of the lateral malleolus. Note the tendon of the peroneus longus muscle.

**Fig 2:**
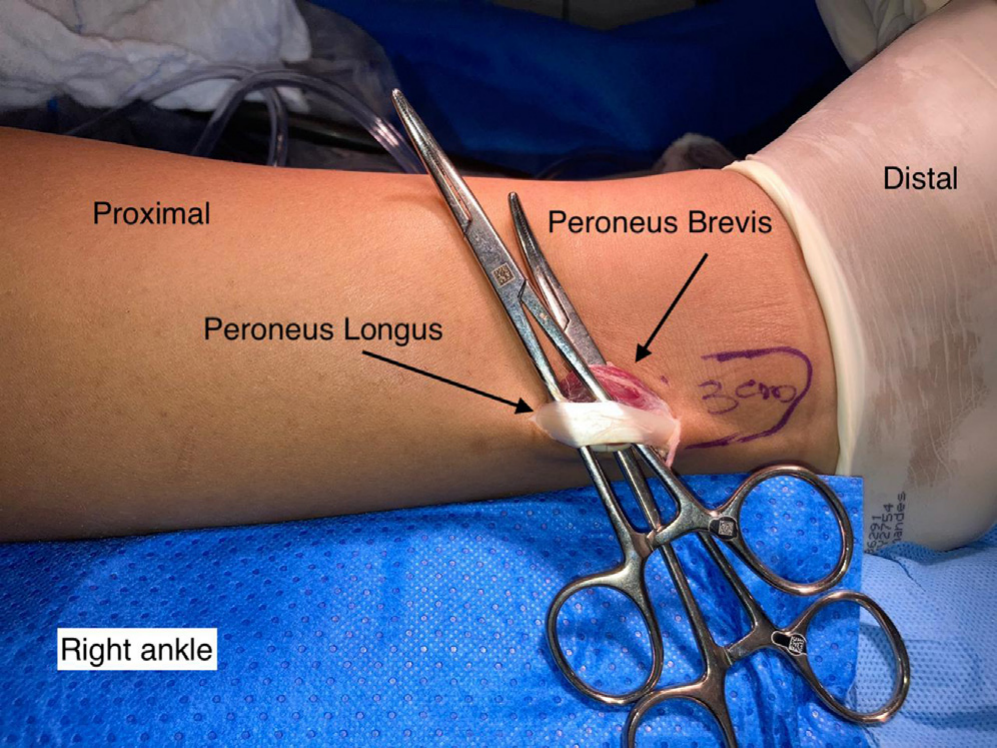
This photo shows the right ankle after the lateral ankle incision was made to remove the peroneus longus tendon graft. A line delimiting the lateral malleolus was drawn. An incision was made starting 3 cm proximal to the most distal point of the lateral malleolus. At this moment, the tendons of the peroneus longus and peroneus brevis muscles are identified. The two tendons are individualized so that the correct union is performed with suture.

**Fig 3:**
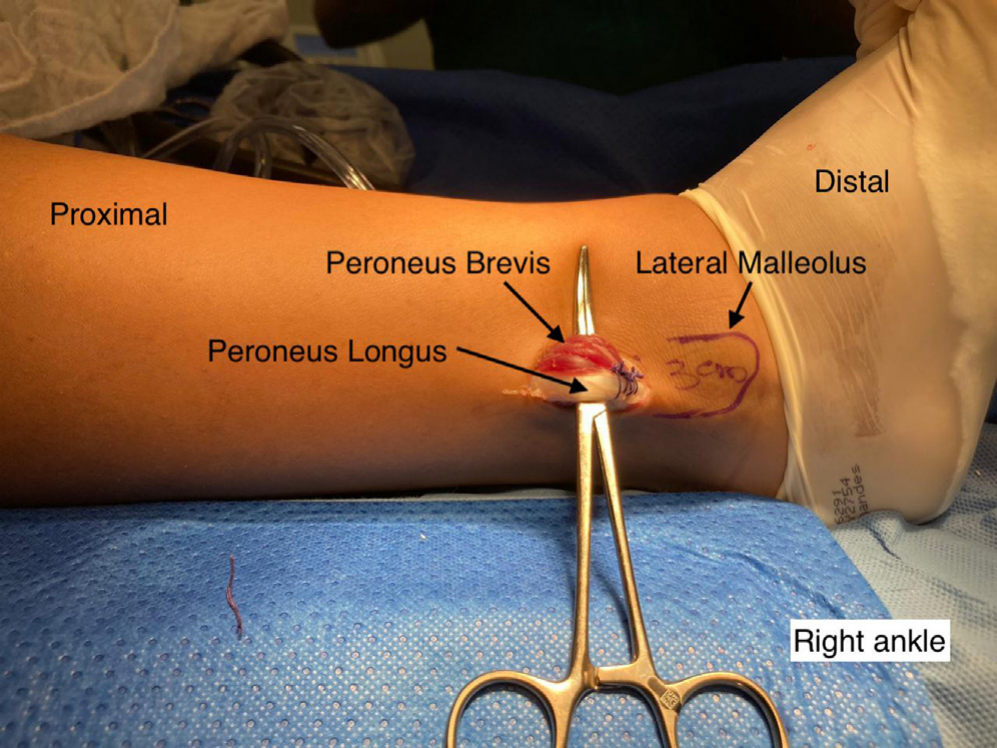
This photo shows the right ankle after the lateral incision was made to remove the peroneus longus tendon graft. A line delimiting the lateral malleolus was drawn. An incision was made starting 3 cm proximal to the most distal point of the lateral malleolus. After identifying the peroneus longus and peroneus brevis tendons, the two tendons are sutured in the most distal region of the incision using simple stitches with 1.0 Vicryl thread (Ethicon, Somerville, NJ).

**Fig 4:**
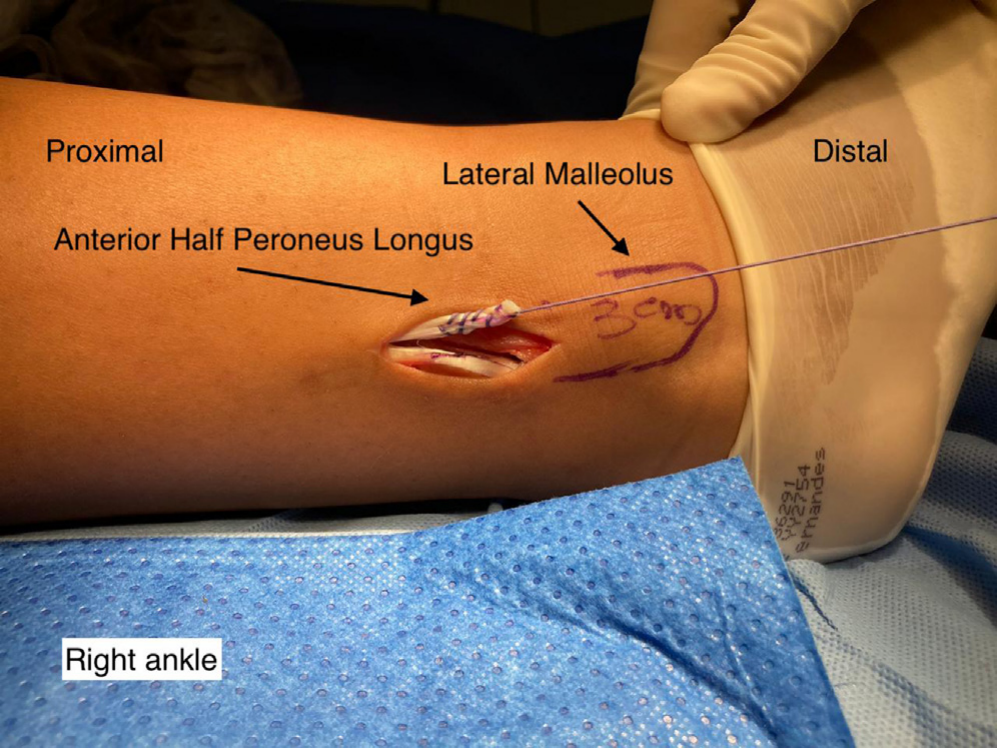
This photo shows the right ankle after the lateral incision was made to remove the peroneus longus tendon graft and the suture between the peroneus longus and peroneus brevis tendons. A line delimiting the lateral malleolus was drawn. After identifying the peroneus longus and peroneus brevis tendons and suturing them in the most distal region of the incision using simple stitches with Vicryl 1.0 thread (Ethicon, Somerville, NJ), a longitudinal incision is made, splitting the peroneus longus tendon into two halves. The anterior half is repaired with a continuous stitch performed with 1.0 Vicryl thread (Ethicon, Somerville, NJ).

A single longitudinal incision of ∼3 cm is made in the posterolateral region of the fibula over the PL (Fig 1). The incision is made starting 3 cm proximal to the most distal point of the lateral malleolus, through the skin and subcutaneous tissue. After the subcutaneous tissue is separated, the PL is identified and isolated with the aid of a hemostatic forceps (Kelly) after it is distinguished from the peroneus brevis (Fig 2). Both peroneal tendons are then brought together in the most distal region of the incision using single sutures with no. 1-0 Vicryl thread (Ethicon, Somerville, NJ) (Fig 3). After the tendons are unified with sutures, the PL is divided in anterior and posterior with a scalpel, the anterior half is repaired with no. 1-0 Vicryl thread (Ethicon, Somerville, NJ) (Fig 4) and removed to its proximal insertion with the aid of a tenotome.

### Graft Preparation

**Fig 5:**
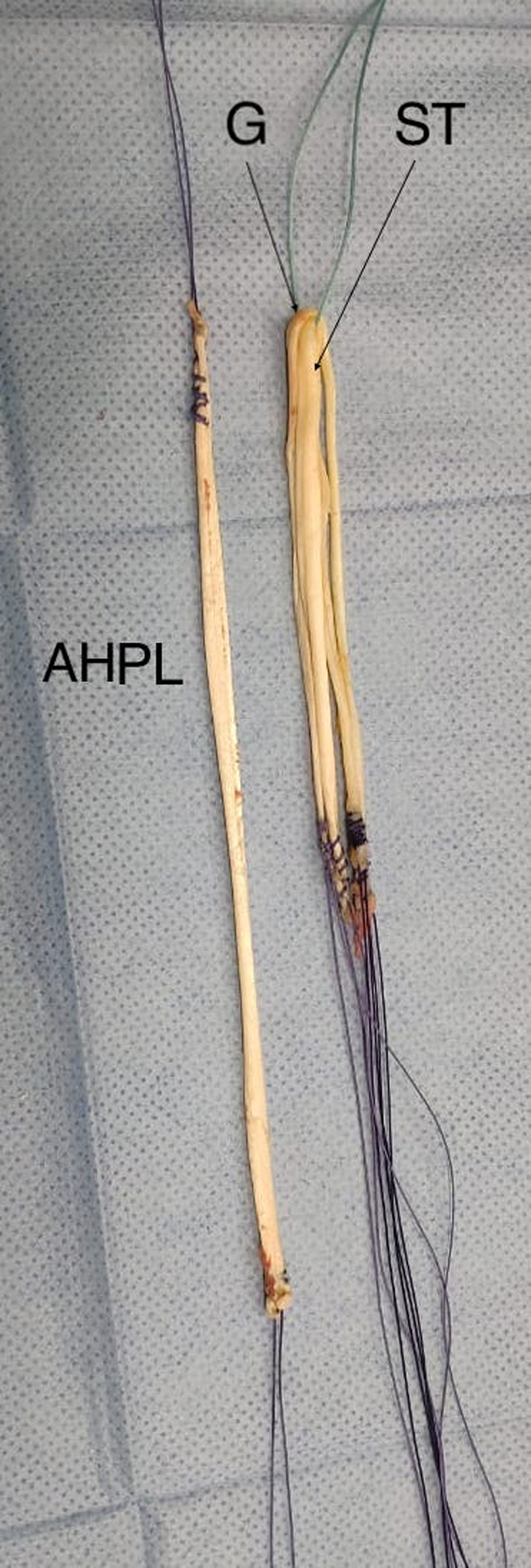
On the back table the grafts are observed. The anterior half of the peroneus longus (AHPL) was prepared with continuous sutures at both ends performed with 1.0 Vicryl thread (Ethicon, Somerville, NJ). The hamstrings (G and ST) were also prepared with continuous sutures at both ends and folded in half forming a traditionally used quadruple graft.

**Fig 6:**
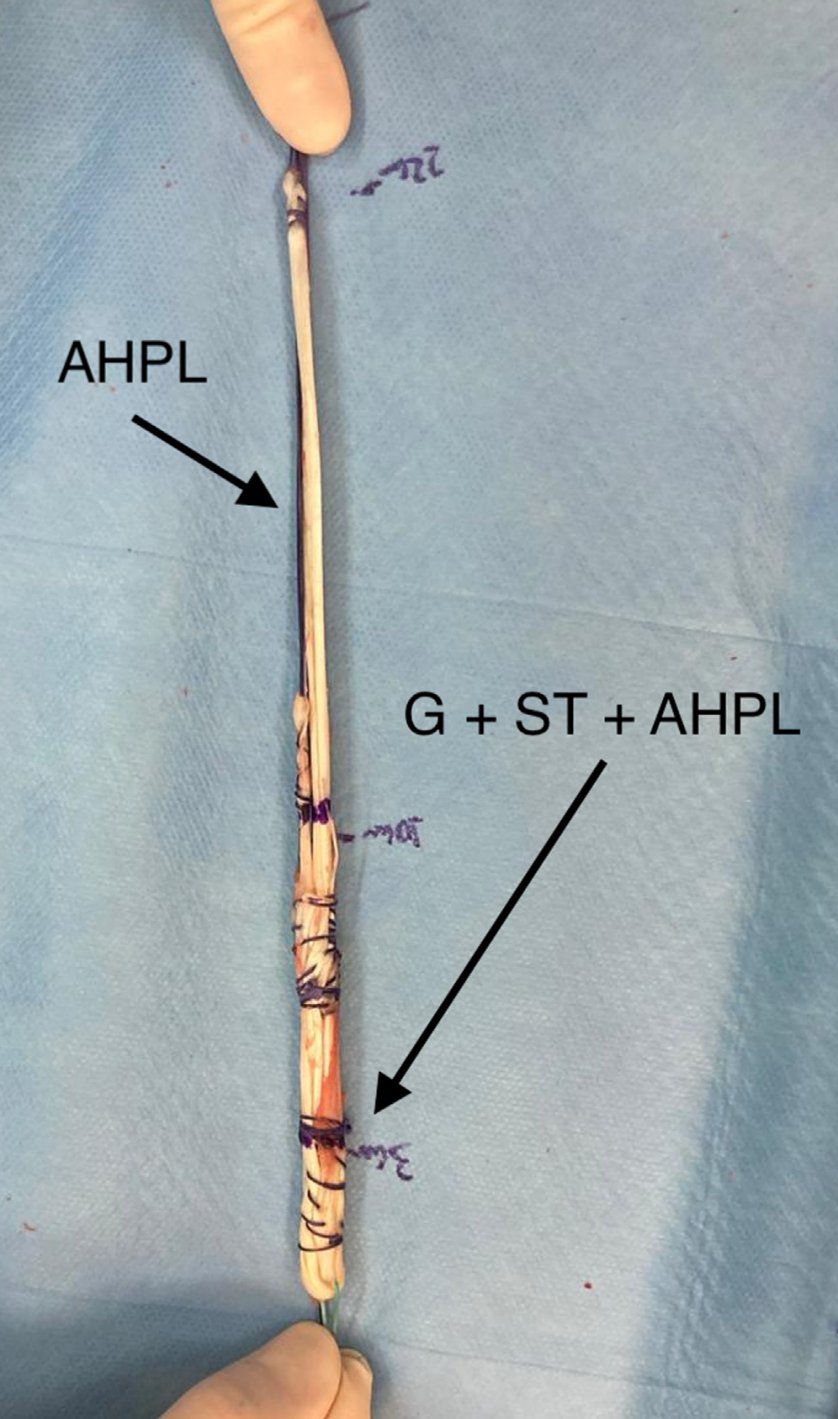
On the back table, the anterior half of the peroneus longus (AHPL) was prepared with continuous sutures at both ends performed with 1.0 Vicryl thread (Ethicon, Somerville, NJ). The hamstrings (G and ST) were also prepared with continuous sutures at both ends and folded in half forming a traditionally used quadruple graft. The AHPL is then incorporated into the quadruple graft forming a quintuple graft in the most proximal region and a single graft in the distal region.

The STG grafts are folded to form a single quadruple graft. Subsequently, the PL graft is incorporated into the STG grafts without folding, forming a quintuple graft in the most proximal region and a single, more distal graft comprising the remainder of the length of the PL, as shown in Figs 5 and 6.

### Arthroscopy

Standardized anterolateral and anteromedial portals are used. We opt for a high anterolateral portal close to the patellar edge and flush with the patellar tendon. The anteromedial portal is created with care taken to form a good angle of inclination with the lateral intercondylar wall.

### ACL Tunnels

Reconstruction is performed through anatomic positioning to create the femoral and tibial tunnels. The femoral tunnel is positioned in the anatomical position. Guides (Acuflex Pinpoint; Smith & Nephew, Andover, MA) are used to make the tunnels using the outside-in technique.

### AOL Femoral Tunnel

With the aid of radioscopy, the medial epicondyle is identified, in the lateral view. At this point, a guidewire (Smith & Nephew) is passed through the femur, starting at the medial epicondyle anterior portion, in the anterior direction, and a bone tunnel is constructed under radioscopic visualization.

### Passage and Fixation

**Fig 7:**
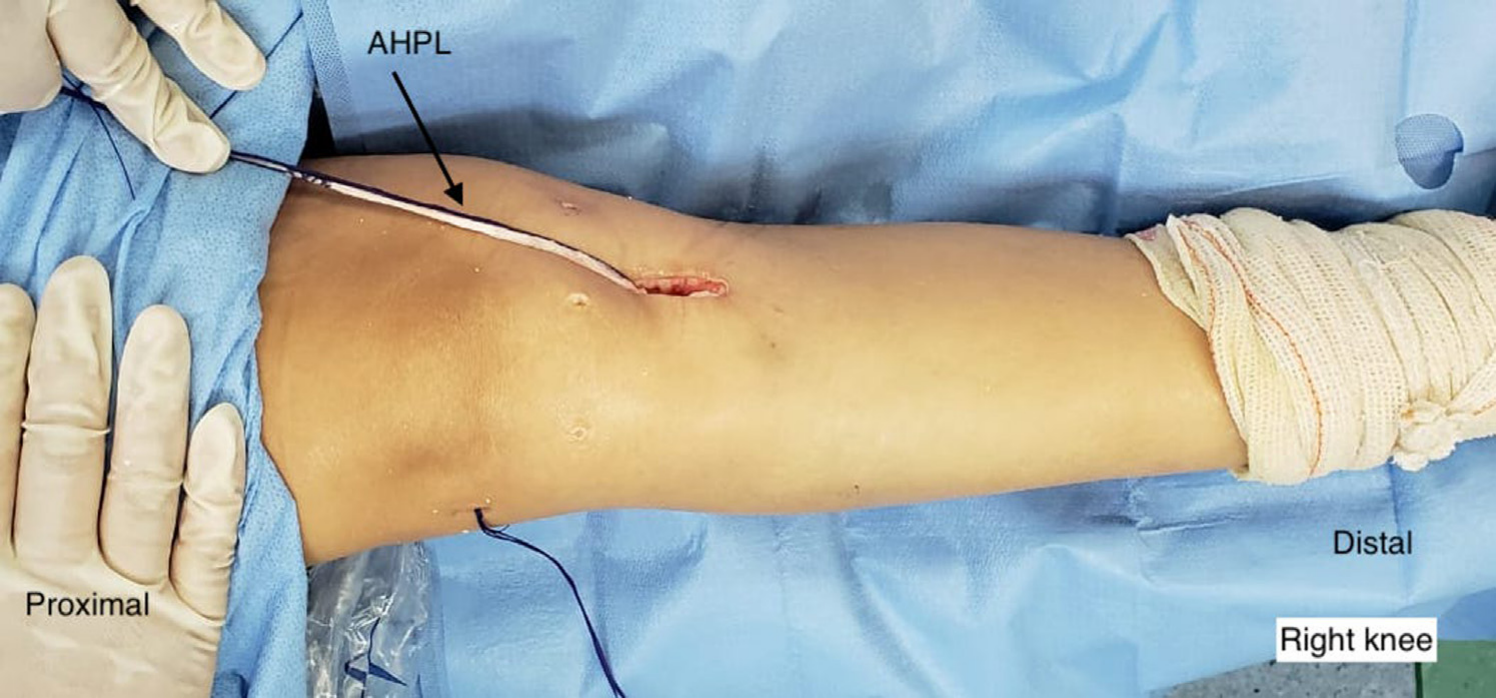
This photo shows the right knee after the graft has been fixed in the tunnels prepared for the anterior cruciate ligament with interference screws (Biosure; Smith & Nephew, Andover MA). It is possible to see the incision through which the hamstring grafts were removed and where the tibial tunnel was drilled. The distal part of the graft is then directed through the subcutaneous tissue from the medial region to the point of attachment on the femur.

**Fig 8:**
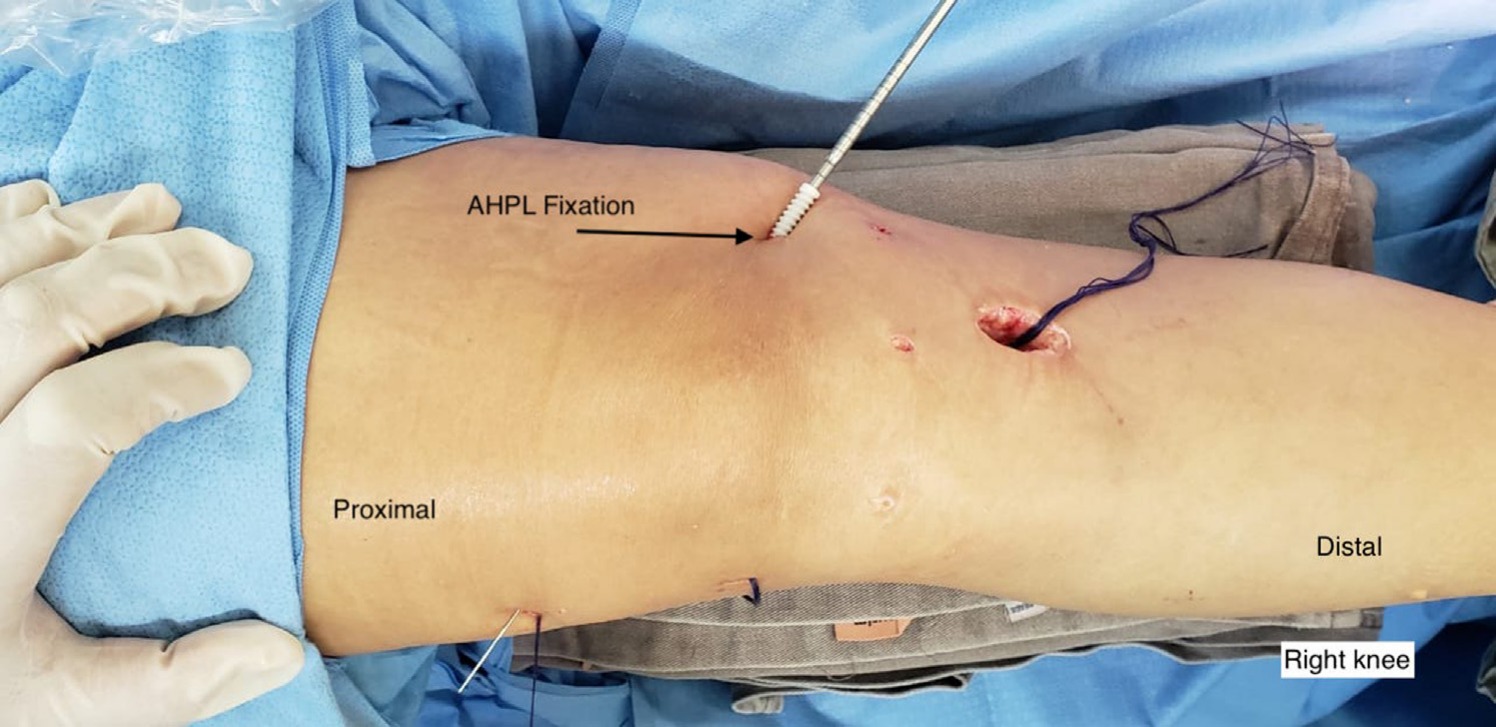
This photo shows the right knee after the graft has been fixed in the tunnels prepared for the anterior cruciate ligament with interference screws (Biosure; Smith & Nephew, Andover MA). The distal part of the graft is then directed through the subcutaneous tissue from the medial region to the point of attachment on the femur. The ideal point of fixation on the femur is guided by fluoroscopy, and the single graft is fixed under traction, neutral rotation, and extension with an interference screw (Biosure).

The graft is pulled through the tibial tunnel and passed through the femoral tunnel with the aid of a no. 5.0 Ethibond thread (Ethicon, Somerville, NJ). The single portion of the graft remains out of the tibial tunnel until the quintuple graft portion occupies both tunnels. The graft is then pulled, and an interference screw (Biosure; Smith & Nephew, Andover MA) is fixed to the femur. The next step is to fix the inferior end of the quintuple graft to the tibia after pre-tensioning. Fixation is performed with the graft tensioned and the knee in total extension and after performance of the posterior drawer maneuver. After tibial fixation, the remaining PL is passed through the subcutaneous, in the medial side, and is passed through the Anterior Oblique Ligament in the femur (Fig 7). The ligament is fixed under traction, neutral rotation and extension with an interference screw (Biosure; Smith & Nephew). (Fig 8, Video 1). Pearls and pitfalls (Table 1), as well as advantages and disadvantages (Table 2) of this technique are summarized.

**Table 1:** Pearls and Potential Pitfalls of ACL and AOL Reconstruction Using Hamstrings and Peroneus Longus’ Anterior Half Grafts

Pearls	Pitfalls
It is important to suture the tendons of the peroneus longus and brevis muscles with knots that do not damage the superficial tissues to avoid the formation of granulomas.	Tightening the screw deeply past the cortical bone may cause loosening of the fixation causing failure.
Guiding the positioning of the femoral tunnel, in which the AOL will be fixed with fluoroscopy is essential for a good result.	Performing the fixation in the femoral tunnel for the AOL in extension and neutral rotation is important for good results.
The positioning of the tunnels for ACL reconstruction with extra joint reinforcement is exactly the same as for ACL reconstruction combined with the AOL.	

ACL, anterior cruciate ligament; AOL, anterior oblique ligament.

**Table 2:** Advantages and Disadvantages of ACL and AOL Reconstruction Using Hamstrings and Peroneus Longus’ Anterior Half Grafts

Advantages	Disadvantages
Simple to perform and few steps are added to the typical ACL reconstruction technique	More graft fixation devices are needed when compared to the traditional outside-in technique.
Use of the quintuple graft for ACL reconstruction increases the graft diameter.	It is necessary to use a graft outside the knee site.
Greater rotational stability occurs in the knee when extra-articular reinforcement is performed.	

ACL, anterior cruciate ligament; AOL, anterior oblique ligament.

## Discussion

Football players sustain the greatest number of ACL injuries (53% of the total) with skiers and gymnasts also at high risk.^1^ Despite outcome studies after ACLR in elite athletes showing a high return-to-sport rate,^8^ recent research has indicated that up to 35% of athletes specifically don’t return to their previous levels of preinjury function within 2 years from ACLR. Furthermore, reinjury to the same surgical knee following an ACL injury has been reported often. ACL rerupture rate has remained between 4.5% and 11%, but it may be significantly higher, even up to 50% within the first postoperative year.

Adequate treatment of all concomitant injuries, aiming at joint stability, and extra-articular ligament reinforcement can lead to better results. Lateral extra-articular procedures (LEAPs) in the anterior cruciate ligament injured knee were widely abandoned in the 1990s but have seen a recent resurgence. The LEAPs associated with ACL reconstructions seem to have a great importance in the surgery survival. In a recent study, by the Santi Group, patients who underwent combined ACL+ALLR (anterolateral ligament reconstruction) experienced significantly better long-term ACL graft survivorship, lower overall rates of reoperation, and no increase in complications compared with patients who underwent isolated ACLR. Further, patients who underwent isolated ACLR had a >5-fold increased risk of undergoing revision surgery at a mean follow-up of 104.3 months.^8^ Tibial internal rotation is controlled mostly by the lateral extra-articular structures, which are the lateral capsule and the iliotibial band. The ALL is a capsular thickening, which has, similar to the deep MCL, only a secondary role in restraining the anterior subluxation of the lateral tibial plateau.^8^ But maybe, when the anterolateral quadrant is reinforced, knee stability is improved, and results like the Santi’s are possible.

A question that arises: Would the ALL be the answer for all?

Most ACL injuries occur without physical contact between athletes—referred to as noncontact ACL injuries^5^—and most occur through a noncontact mechanism of injury in sports, in which sudden deceleration, landing, and pivoting maneuvers are repeatedly performed. Della Villa and colleagues^5^^,^^9^ showed that biomechanically, the knee valgus represented 81% and 88% in ACL lesions in male and female professional soccer players, having a foot external rotation of 66% and 68%, respectively.

By the Theory of Tibial Quadrants,^6^ the knee’s anteromedial quadrant is the responsible for valgus and external rotational control, and the reinforcement of this area shows, in vitro, improvement of the knee stability, controlling the anteromedial rotational instability (AMRI).^10^ The deep medial collateral ligament (MCL) is positioned in the anteromedial quadrant, in the same position. Nowadays, the interest on the knee medial anatomy is growing, and the surgical techniques are being improved,^11^ and the anteromedial quadrant is being revisited by different authors.^11^^,^^12^

Our group believes that, in almost all ACL ruptures, the extra-articular reinforcement is important, and in the future, it will be routinely done. But we also believe that the anterolateral reinforcement is not the answer to all cases. The patients that don’t have grade 2 pivot shift, the patients that have a mild medial instability (not treated in past), and the patients who have a history of valgus and external rotational trauma, can benefit from anteromedial reinforcement in the AOL topography.

And for this, the use of peroneal longus tendon, with hamstrings, can be a good and secure option.^4^^,^^13^^,^^14^

## Supplementary Data

ICMJE author disclosure forms

Video 1 The procedure is performed with the patient in the supine position on the surgical table. Harvest of the tendons of the semitendinosus and gracilis muscles is preferentially performed in the injured limb. A single longitudinal incision of approximately 3 cm is made in the posterolateral region of the fibula over the peroneus longus. After the subcutaneous tissue is separated, the peroneus longus is identified and isolated with the aid of a hemostatic forceps. Both peroneal tendons are then brought together in the most distal region of the incision using single sutures with Vicryl thread. After the tendons are unified with sutures, the peroneus longus is divided in anterior and posterior with a scalpel, the anterior half is repaired with Vicryl thread and removed to its proximal insertion with the aid of a tenotome. The hamstrings grafts are folded to form a single quadruple graft. Subsequently, the peroneus longus graft is incorporated into the hamstrings grafts without folding, forming a quintuple graft in the most proximal region and a single, more distal graft, comprising the remainder of the length of the peroneus longus. Reconstruction is performed through outside-in anatomic positioning to create the femoral and tibial tunnels. The graft is pulled through the tibial tunnel, and an interference screw is fixed to the femur. The next step is to fix the inferior end of the quintuple graft to the tibia after pretensioning. After tibial fixation, the remaining PL is passed through the subcutaneous, in the medial side, and is passed through the anterior oblique ligament in the femur. With the aid of radioscopy, the medial epicondyle is identified, in the lateral view. At this point, a guidewire is passed through the femur, starting at the medial epicondyle anterior portion, in the anterior direction, and a bone tunnel is constructed. The ligament is fixed under traction, neutral rotation, and extension with an interference screw.
